# Case Report: First report of vonoprazan-associated severe Gly m 4–mediated PFAS anaphylaxis in the era of potent acid suppression

**DOI:** 10.3389/falgy.2026.1893499

**Published:** 2026-08-18

**Authors:** Makoto Nojo, Shintaro Suzuki, Tomoki Uno, Nao Sato, Sae Kawafune, Reira Masuda, Takaya Ebato, Tomohiro Matsunaga, Yoshitaka Uchida, Hironori Sagara, Akihiko Tanaka

**Affiliations:** 1 Division of Respiratory Medicine and Allergology, Department of Internal Medicine, Showa Medical University School of Medicine, Shinagawa City, Tokyo, Japan; 2 Department of Medical Education, Showa Medical University School of Medicine, Shinagawa City, Tokyo, Japan

**Keywords:** anaphylaxis, acid suppression, cofactor, Gly m 4, pollen-food allergy syndrome, potassium-competitive acid blocker, soybean, vonoprazan

## Abstract

**Background:**

Pollen–food allergy syndrome (PFAS) usually causes localized oral symptoms through cross-reactivity between pollen allergens and plant-derived foods. However, the soybean PR-10 allergen Gly m 4 can provoke systemic reactions, including anaphylaxis. Proton pump inhibitors (PPIs) are recognized in PFAS consensus statements as potential cofactors, although acid suppression is not established as a major cofactor for food-induced anaphylaxis overall. Increasingly prescribed potassium-competitive acid blockers (P-CABs), including vonoprazan, provide potent and sustained acid suppression beyond conventional PPIs. To our knowledge, severe PR-10–related PFAS temporally associated with P-CAB therapy has not been previously reported.

**Case presentation:**

A 59-year-old woman had experienced mild oral allergy syndrome–like symptoms, including lip swelling, after soy milk ingestion, while tolerating other soybean products. Two weeks after starting vonoprazan 20 mg for gastroesophageal reflux disease, she developed generalized urticaria, throat discomfort, and dyspnea after dinner containing inadequately cooked soybean sprouts, requiring epinephrine. Three months later, within 15 min after eating ramen containing inadequately cooked soybean sprouts, she developed generalized urticaria and dyspnea, requiring hospitalization and epinephrine. Component-resolved testing showed positive specific IgE to Gly m 4, whereas crude soybean extract, *ω*-5 gliadin, and other food-related allergens were negative. These findings supported Gly m 4–mediated PR-10–related PFAS. Because vonoprazan was the only new factor common to both episodes, it was considered a possible cofactor. Vonoprazan was discontinued, and the patient was advised to avoid soy milk and undercooked soybean sprouts and to be cautious regarding recognized cofactors. Over 6 months of follow-up, no further anaphylaxis occurred, and soybean sprouts were tolerated.

**Conclusion:**

To our knowledge, this is the first report of severe Gly m 4–mediated PFAS anaphylaxis temporally associated with vonoprazan use. Potent P-CAB–mediated acid suppression may theoretically reduce gastric degradation of labile PR-10 allergens and increase systemic exposure to immunologically active Gly m 4, although causality was not proven because oral food challenge with and without vonoprazan was not performed. These findings suggest that, in PR-10–related PFAS, medication review and anticipatory counseling regarding potent acid suppression, particularly P-CAB therapy, may be important in patients with PFAS, even when previous symptoms have been limited to mild oral reactions.

## 1 Introduction

Pollen–food allergy syndrome (PFAS) is an IgE-mediated immediate-type allergic reaction caused by ingestion of plant-derived foods cross-reactive with pollen allergens in sensitized individuals. In Japan, the prevalence of pollinosis has increased from 19.6% to 42.5% over the past two decades (1), suggesting a parallel rise in PFAS (2). Although PFAS is often limited to oropharyngeal symptoms, systemic reactions can occur, and their severity may be influenced by cofactors such as seasonal pollen exposure, physical activity, medications, and the degree of food processing.

Among the allergen families involved in PFAS, the pathogenesis-related protein 10 (PR-10) family is particularly important. PR-10 allergens include Bet v 1 homologues and related proteins associated with birch- and alder-pollen sensitization (3). Gly m 4, the soybean PR-10 homolog, is a Bet v 1–homologous protein and is closely associated with soybean-related PFAS in patients sensitized to Fagales pollens (4, 5).

Although PR-10 allergens are generally heat- and digestion-labile, Gly m 4–related PFAS is clinically notable because systemic reactions occur more frequently than in many other PR-10–related PFAS phenotypes; minimally processed or briefly heated soybean products, including soy milk and soybean sprouts, can provoke anaphylaxis (2, 3).

**Figure 1:**
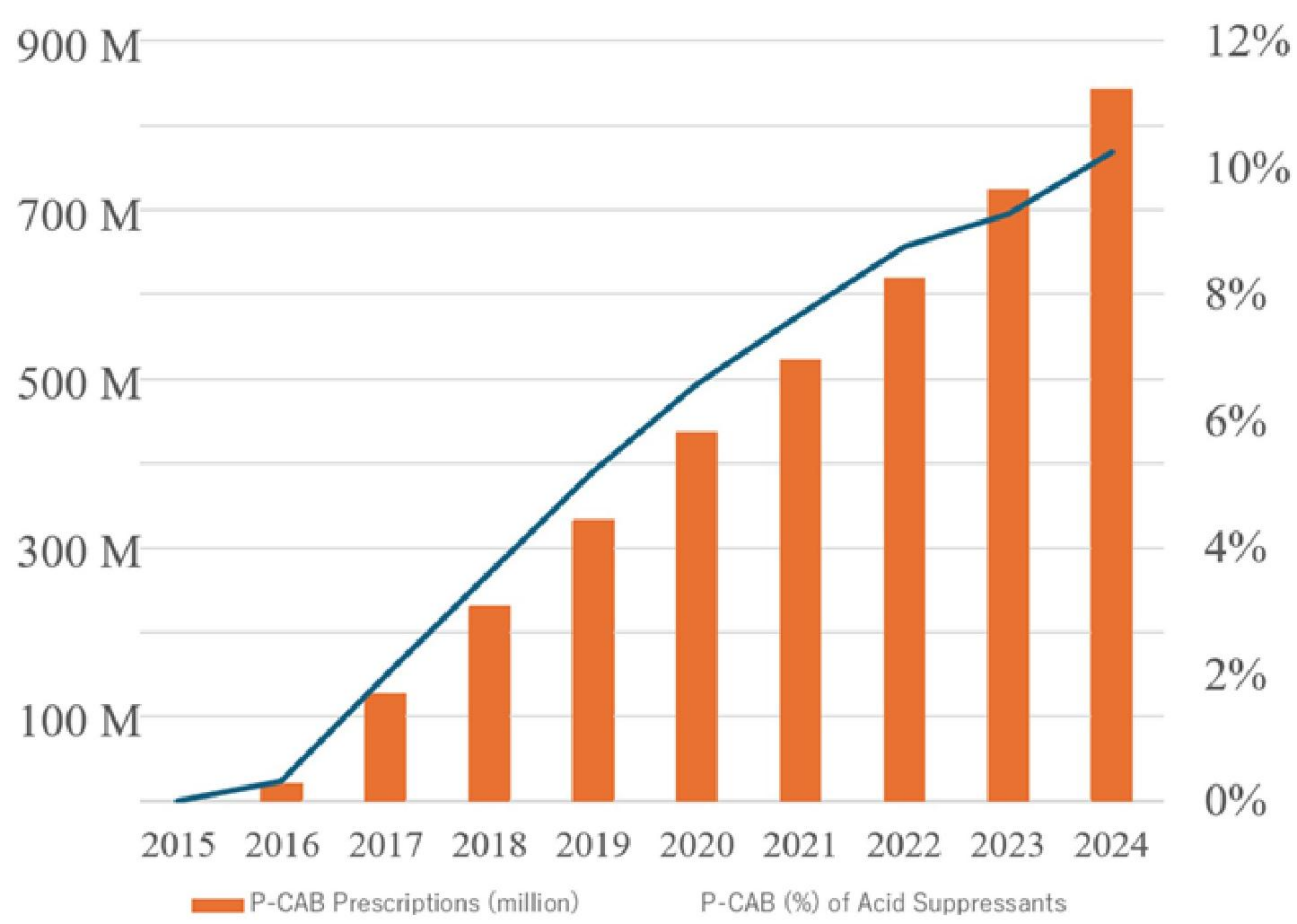
Trends in potassium-competitive acid blocker (P-CAB) prescriptions in Japan. P-CAB, potassium-competitive acid blocker. Bars represent the annual number of P-CAB prescriptions in Japan, and the line represents the proportion of P-CABs among all acid-suppressive agents. The numerator was defined as annual prescriptions for P-CABs, and the denominator was defined as annual prescriptions for acid-suppressive agents included in the authors' extraction from the NDB Open Data. Data source: Ministry of Health, Labour and Welfare, Japan. The 10th NDB Open Data, prescribed medicines, based on fiscal year 2024 claims (12). Accessed Oct 2025.

Acid-suppressive therapy is not usually regarded as a major cofactor in food allergy overall, but PFAS caused by heat- and pepsin-labile allergens may be an exception (6, 7). Reduced gastric acidity and peptic activity may increase the amount of immunologically active allergen reaching the intestine (8). Recent PFAS consensus statements list proton pump inhibitors among potential facilitators of systemic reactions, and a multicenter study of labile plant-food allergy found PPI use more frequently in systemic reactors than in patients with oral allergy syndrome alone (9). Potassium-competitive acid blockers (P-CABs), including vonoprazan, provide more rapid, potent, and sustained acid suppression than conventional PPIs and are increasingly used in Japan. In our analysis of Japanese national prescription data, P-CAB accounted for approximately 10% of acid-suppressant prescriptions in 2024 (Figure 1)^,^ with early uptake also reported among gastroenterology prescribers in the United States. Potent acid suppression with P-CABs may therefore be a biologically plausible, but unproven, cofactor in PR-10–related PFAS.

We report, to our knowledge, the first case of Gly m 4–related PFAS with recurrent anaphylaxis after ingestion of briefly heated soybean sprouts temporally associated with vonoprazan treatment. This case highlights potent acid suppression by P-CABs as a biologically plausible and clinically relevant candidate cofactor in PR-10–related PFAS, particularly as P-CAB prescribing continues to increase.

## Case presentation

The patient was a 59-year-old woman who had previously experienced mild oral allergy syndrome (OAS)-like symptoms, including lip swelling, after soymilk ingestion, but continued to consume other soybean products without restriction. Vonoprazan, a potassium-competitive acid blocker, had been initiated at a dose of 20 mg once daily for mild gastroesophageal reflux disease (GERD).

The first episode occurred in September, 2 weeks after the initiation of vonoprazan. On that day, the last dose of vonoprazan had been taken approximately 9 h before dinner. At 6:00 PM, ate dinner that included soybean sprouts prepared as part of stir-fried meat and vegetables; the soybean sprouts had been pan-fried over medium heat for approximately 5 min, a common cooking method that leaves the vegetables relatively crisp. At approximately 7:00 PM, she began light walking. At approximately 7:20 PM, she developed generalized urticaria corresponding to Sampson grade 2, throat discomfort corresponding to grade 1, and dyspnea. She was diagnosed with anaphylaxis at a local hospital and improved after epinephrine administration.

**Figure 2:**
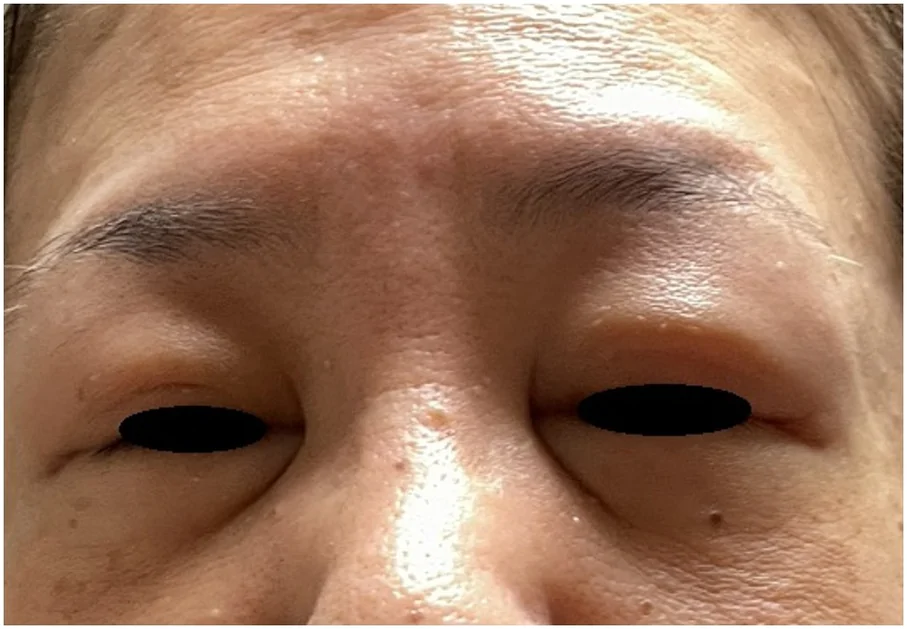
Facial angioedema during the second anaphylactic episode. Clinical photograph obtained during the patient's second anaphylactic episode, showing prominent bilateral periorbital and facial angioedema. The episode was accompanied by generalized urticaria, predominantly involving the face, and respiratory symptoms.

The second episode occurred in December, 3 months after the first episode, while she was still receiving vonoprazan 20 mg once daily. On that day, the last dose of vonoprazan had been taken approximately 4 h before lunch. At 1:00 PM, she ate ramen containing soybean sprouts that had been briefly boiled for approximately 1–2 min as a topping. Approximately 20 min later, she developed generalized urticaria with marked facial angioedema, corresponding to Sampson grade 2, and dyspnea. No exercise or other apparent physical exertion preceded this episode. She required hospitalization and treatment including epinephrine. She was referred to our department for evaluation of recurrent anaphylaxis (Figure 2).

At the initial visit to our department, she was asymptomatic, and physical examination was unremarkable. Total IgE was 151 IU/mL. Specific IgE testing was positive for alder pollen (class 3; 9.57 UA/mL) and Gly m 4 (class 3; 5.66 UA/mL). Bet v 1 and soy storage proteins, including Gly m 5, Gly m 6, and Gly m 8, were not assessed. serum tryptase was not measured. Specific IgE to crude soybean extract and selected allergens suspected from the dietary history was negative. Additional component-resolved and extract-based tests performed for differential diagnostic purposes, including *ω*-5 gliadin, gluten, Jug r 1, Ana o 3, Ara h 2, *α*-lactalbumin, *β*-lactoglobulin, casein, and Hev b 6.02, were all negative.

Potential cofactors were systematically reviewed, including exercise, bathing or heat exposure, alcohol intake, intercurrent infection, hormonal factors, medications such as oral contraceptives, nonsteroidal anti-inflammatory drugs/aspirin, acid-suppressive agents, antihypertensive drugs, excessive stress, sleep deprivation, and seasonal pollen exposure. Apart from light postprandial walking during the first episode and ongoing vonoprazan use during both episodes, no consistent cofactors were identified. Although exercise could not be completely excluded as a contributing factor in the first episode, the second episode occurred without preceding exercise or physical exertion, arguing against exercise as the sole explanation.

The first and second episodes occurred in September and December, respectively, both outside the local alder/birch-related pollen season, which generally extends from January to May and peaks from February to April. Thus, a temporal association between the anaphylactic episodes and seasonal pollen exposure was considered unlikely.

Based on the history of soy milk–induced OAS-like symptoms, recurrent anaphylaxis after ingestion of briefly cooked soybean sprouts, sensitization to alder pollen, and positive specific IgE to Gly m 4, the patient was diagnosed with soybean sprout–triggered anaphylaxis associated with Gly m 4-related, PR-10–mediated PFAS. Because Bet v 1 and soy storage protein components were not assessed, the absence of primary Bet v 1 sensitization or concomitant sensitization to soy storage proteins was not directly demonstrated. Nevertheless, the integrated clinical and immunologic profile was considered most consistent with Gly m 4-related PFAS.

The temporal relationship between vonoprazan exposure and the two anaphylactic episodes raised suspicion that vonoprazan had acted as a clinically relevant cofactor to symptom exacerbation. Because the patient's GERD was mild, vonoprazan was discontinued. She was initially advised to avoid soy milk and raw or briefly heated soybean sprouts and to exercise caution regarding recognized PFAS cofactors, including exercise, alcohol, fasting, and nonsteroidal anti-inflammatory drugs.

After discontinuation of vonoprazan, soybean sprouts were reintroduced gradually in daily life. She subsequently tolerated soybean sprouts prepared with comparable cooking methods, including stir-fried and briefly boiled preparations, without recurrence. At the latest follow-up, she was eating soybean sprouts approximately once per month, including during and after the subsequent alder/birch-related pollen season, and remained free from urticaria, angioedema, respiratory symptoms, and anaphylaxis.

## Discussion

The clinical significance of this case lies in its demonstration of the possibility that potent gastric acid suppression by a potassium-competitive acid blocker (P-CAB) may act as a cofactor lowering the threshold for systemic reactions in Gly m 4-related pollen-food allergy syndrome (PFAS). Before initiation of vonoprazan, the patient had experienced only oral symptoms after soy milk ingestion and had tolerated soybean sprouts. However, after initiation of vonoprazan, she developed two episodes of anaphylaxis requiring epinephrine after ingestion of soybean sprouts. After discontinuation of vonoprazan, she subsequently tolerated soybean sprouts prepared in a similar manner on multiple occasions, including during and after the subsequent alder/birch-related pollen season. Although this clinical course does not prove causality, the recurrence during vonoprazan exposure and the absence of recurrence after discontinuation support the possibility that vonoprazan acted as a clinically meaningful cofactor.

Gly m 4 is a Bet v 1-homologous PR-10 allergen and is generally considered heat- and digestion-labile; however, Gly m 4-related soybean allergy has been reported to cause not only oral symptoms but also systemic reactions (2, 3, 10). In addition, Gly m 4 may retain IgE-binding activity after processing and heating, while remaining susceptible to degradation by gastric acid and pepsin (8, 11). In the present case, the patient tolerated soybean sprouts prepared under similar cooking conditions after discontinuation of vonoprazan, making it difficult to explain the reactions by the degree of heating alone. Rather, potent acid suppression may have reduced gastric degradation of the allergen and increased the amount of immunologically active Gly m 4 reaching the intestine.

Other cofactors also require careful interpretation. In the first episode, light walking occurred after ingestion, and cofactor-dependent food allergy cannot be completely excluded. However, the second episode occurred without apparent exercise, and no typical exacerbating factors, such as bathing, alcohol intake, nonsteroidal anti-inflammatory drugs, infection, or sleep deprivation, were identified (6, 7). Furthermore, the episodes occurred outside the main alder/birch pollen season, and after discontinuation of vonoprazan, systemic reactions after soybean sprout ingestion did not recur even under circumstances involving similar walking, including during and after the pollen season. These clinical findings are more consistent with acid suppression by P-CAB therapy as the major modifying factor than with exercise or seasonal pollen exposure.

Assessment of cofactors is important in the management of PFAS, and acid-suppressive drugs may also alter allergen exposure by reducing gastric digestion (8). P-CABs provide more rapid, potent, and sustained gastric acid suppression than conventional proton pump inhibitors (PPIs) (9). Therefore, although a difference in clinical risk between P-CABs and PPIs has not been established, P-CABs may have particular mechanistic relevance in PFAS involving acid- and pepsin-labile allergens. Furthermore, P-CAB prescriptions have been increasing in Japan, and if similar agents become more widely used in other countries, the risk of unexpected systemic reactions in patients with PR-10-related PFAS may become a broader clinical issue (10, 12) (Figure 1). Therefore, when patients with PFAS to PR-10-related plant foods, including soybean, develop systemic reactions that are more severe than their previous symptoms, clinicians should assess the use of acid-suppressive drugs, including PPIs and P-CABs, in addition to exercise, nonsteroidal anti-inflammatory drugs, alcohol intake, fasting, and infection (6, 7). Particularly in the context of P-CAB therapy, explaining to patients with PR-10-related PFAS, even those whose previous symptoms were mainly confined to the oral cavity, that potent acid suppression may lower the reaction threshold and incorporating this information into patient education may provide a new perspective for preventing severe reactions.

The main limitation of this report is that oral food challenge testing with and without vonoprazan was not performed, precluding direct causal inference. In addition, Bet v 1, soybean storage protein components, and serum tryptase were not measured, which limits interpretation of the sensitization profile and underlying mechanism. Nevertheless, the repeated occurrence of reactions after initiation of vonoprazan, the absence of recurrence after discontinuation, the occurrence of a reaction independent of exercise, the weak temporal relationship with the pollen season, and the mechanistic plausibility together suggest that vonoprazan may have acted as a cofactor for Gly m 4-related PFAS anaphylaxis.

## Conclusion

Although causality remains unproven, this first report of severe Gly m 4–mediated PFAS anaphylaxis temporally associated with P-CAB therapy suggests that medication review and counseling regarding potent acid-suppressive therapy, particularly P-CABs, should be incorporated into the management of PFAS involving soy or other labile plant-food allergens.

## Data Availability

The original contributions presented in the study are included in the article; further inquiries can be directed to the corresponding author.
